# Conjoined Twin

**Published:** 2012-06-19

**Authors:** Firoozeh Ahmadi, Nahid Keramat, Hadieh Haghighi

**Affiliations:** Department of Reproductive Imaging, Reproductive Biomedicine Research Center, Royan Institute for Reproductive Biomedicine, ACECR, Tehran, Iran

**Fig 1:**
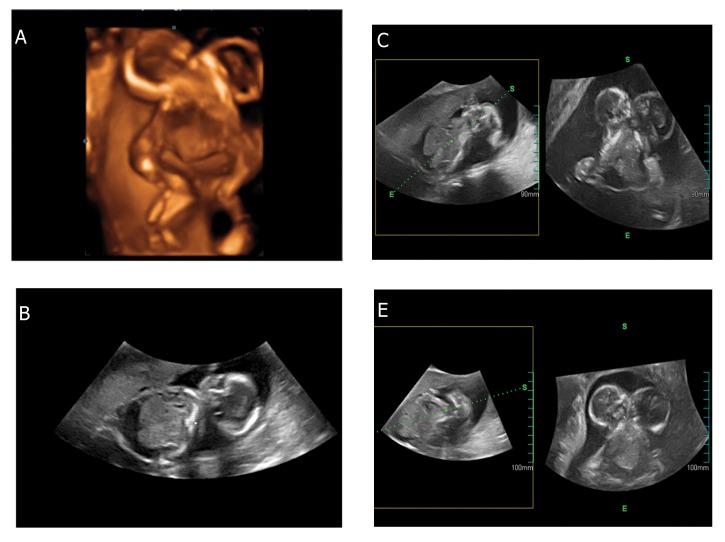
Ultrasound of conjoined twins (CT). These individuals share an anterior connection of the trunk at the thorax and the abdomen. Abdomen of both fetuses was notable for moderate ascites. The pelvic areas were separate with four kidneys and two bladders.

We present a conjoined twin (CT) case of pregnancy in a 31-year-old woman (gravida 2, para 1) who came for her first routine ultrasound at 18 weeks of gestation. She conceived by spontaneous conception. The patient had a healthy child from a previous pregnancy. The initial ultrasound revealed two fetuses with a fused thorax and abdomen, two spines with an unusual extension, one beating heart, shared liver across the fetuses, four kidneys, two bladders, four normally formed arms and legs, and the pelvic were separate with two female external genitalia (Fig 1). The cord had multiple vessels and the abdomen of both fetuses was notable for moderate ascites. With a definite diagnosis of thoracoomphalopagus type of CT that is incompatible with life, the parents decided to terminate the pregnancy.

The parents did not give permission to publish any picture of the twins after termination.

Conjoined fetuses are a rare phenomenon of a monochorionic, monoamniotic twin when the embryo divides at 13-15 days from conception. The incidence ranges from 1/50000-1/100000 live births (1). Female fetuses are more commonly affected, as the ratio of female to males is 3:1, particularly in the thoracopagus type (1). No association with maternal age, race, parity, or heredity has been observed, and the recurrence risk is negligible.

CT are classified according to the anatomical site of union with suffix pagus meaning fixed. The most frequent ventral union is the thoracopagus. Fusion of the abdomen is called omphalopagus and fusion of the thorax and abdomen is known as a thoraco-omphalopagus.

An early ultrasound finding suggestive of CT is the bifid appearance of the fetal pole. The ultras. ound criteria for diagnosis of CT consist of :

- Bifid appearance of a 1^st^ trimester fetal pole (V- or Y- shaped twin pregnancy)
- The heads and bodies of the twins are at the same level
- Single amniotic cavity with no dividing amniotic membranes
- Fetus inseparable with fixed position even after maternal movement
- More than three vessels in the cord
- Single heart
- Unusual extension of the spines
- Unusual proximity of the extremities (2-4).

The presence of these sonographic signs depends on the different types of CT. When amonochorionic and monoamniotic pregnancy is suspected, the presence of these signs must be considered. Two top differential diagnoses are twin reverse arterial perfusion (TRAP) and monoamniotic twin (5). Prognosis depends on the location and length of the fusion, the presence of vital organs such as the liver and heart in both twins, and the presence of associated anomalies. Cesarean section is the recommended method of delivery and surgical separation is necessary.
